# Forsythiasides: A review of the pharmacological effects

**DOI:** 10.3389/fcvm.2022.971491

**Published:** 2022-07-25

**Authors:** Hong-Xuan Yang, Qiu-Ping Liu, Yan-Xi Zhou, Yu-Ying Chen, Pei An, Yi-Zhuo Xing, Lei Zhang, Min Jia, Hong Zhang

**Affiliations:** ^1^Institute of Interdisciplinary Integrative Medicine Research, Shanghai University of Traditional Chinese Medicine, Shanghai, China; ^2^State Key Laboratory of Characteristic Chinese Medicine Resources in Southwest China, College of Pharmacy, Chengdu University of Traditional Chinese Medicine, Chengdu, China; ^3^Library, Chengdu University of Traditional Chinese Medicine, Chengdu, China; ^4^Yueyang Hospital of Integrated Traditional Chinese and Western Medicine, Shanghai University of Traditional Chinese Medicine, Shanghai, China; ^5^Department of Chinese Medicine Authentication, School of Pharmacy, Naval Medical University, Shanghai, China

**Keywords:** forsythiasides, *Forsythia suspensa*, cardiovascular protection, anti-inflammation, anti-oxidation

## Abstract

Forsythiasides are a kind of phenylethanol glycosides existing in *Forsythia suspensa* (Thunb.) Vahl, which possesses extensive pharmacological activities. According to the different groups connected to the nucleus, forsythiasides can be divided into A-K. In recent years, numerous investigations have been carried out on forsythiasides A, B, C, D, E, and I, which have the effects of cardiovascular protection, anti-inflammation, anti-oxidation, neuroprotection, et al. Mechanistically, forsythiasides regulate toll-like receptor 4 (TLR4)/myeloid differentiation factor 88 (MyD88)/nuclear factor kappaB (NF-κB), nuclear factor-erythroid 2-related factor 2 (Nrf2)/heme oxygenase-1 (HO-1) and other signaling pathways, as well as the expression of related cytokines and kinases. Further exploration and development may unearth more treatment potential of forsythiasides and provide more evidence for their clinical applications. In summary, forsythiasides have high development and application value.

## Introduction

Forsythiae Fructus (Chinese name连翘) is the dried fruit of *Forsythia suspensa* (Thunb.) Vahl, a medicinal plant (Oleaceae) widely distributed in Shanxi, Henan, Shaanxi, and other provinces in China. It is commonly used not only in the clinical practice of traditional Chinese medicine (TCM), but also as an important raw material of many Chinese patent drugs due to its traditional efficacies of clearing heat and detoxification, detumescence and dispersing knot, dispelling wind, and clearing heat. Forsythiae Fructus possesses a variety of pharmacological activities, such as anti-inflammation, antioxidation, anti-virus, and anti-bacteria. Numerous compounds are isolated from *F. suspensa*, including phenylethanol glycosides, lignans, flavonoids, terpeniods, volatile oils, etc. (1–3). Forsythiasides are the main components of phenylethanol glycosides which have the highest content in *F. suspensa*.

**FIGURE 1:**
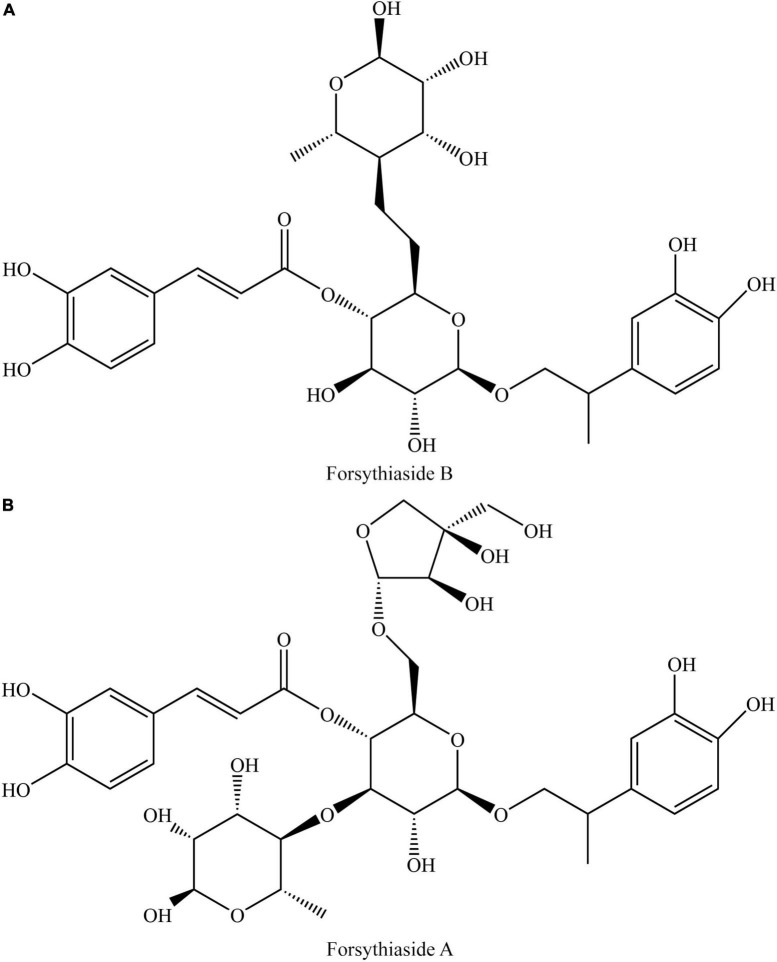
Structures of forsythiaside A **(A)** and forsythiaside B **(B)**.

As the important ingredients for Forsythiae Fructus to exert various pharmacological activities, forsythiasides A-K have been isolated from *F. suspensa* (4–6). Forsythiasides is a kind of glycosides formed by phenylethanol and sugar and have the stem nucleus as shown in Table 1. The glucose can connect with aglycone, rhamnose, xylose, apinose, and caffeoyl. The structures of forsythiasides A-K are displayed in Table 1, and the structures of forsythoside A and B are shown in Figure 1.

**TABLE 1:** The chemical structures of forsythiasides.

Structure code/compound	Structure
Stem nucleus of forsythiasides	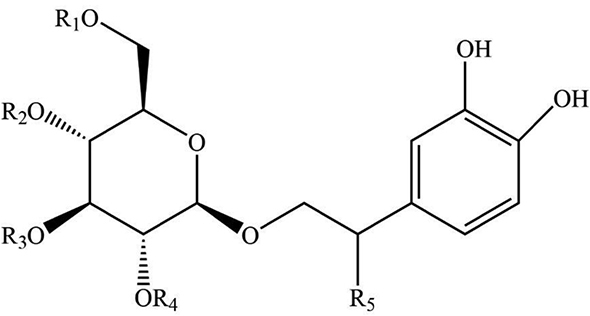
B	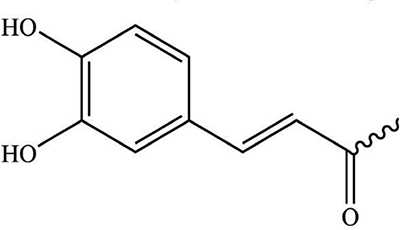
C	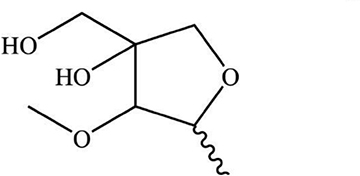
Forsythiaside A	R_1_ = Rha, R_2_ = B, R_3_ = R_4_ = R_5_ = H
Forsythiaside B	R_1_ = Api, R_2_ = B, R_3_ = Rha, R_4_ = R_5_ = H
Forsythiaside C	R_1_ = Rha, R_2_ = B, R_3_ = R_4_ = H, R_5_ = OH
Forsythiaside D	R_1_ = Rha, R_2_ = R_3_ = R_4_ = H, R_5_ = OH
Forsythiaside E	R_1_ = Rha, R_2_ = R_3_ = R_4_ = H, R_5_ = OH
Forsythiaside F	R_1_ = Xyl, R_2_ = B, R_3_ = Rha, R_4_ = R_5_ = H
Forsythiaside G	R_1_ = C, R_2_ = B, R_3_ = Rha, R_4_ = R_5_ = H
Forsythiaside H	R_1_ = Rha, R_2_ = R_3_ = R_5_ = H, R_4_ = B
Forsythiaside I	R_1_ = Rha, R_2_ = R_4_ = R_5_ = H, R_3_ = B
Forsythiaside J	R_1_ = Xyl, R_2_ = R_3_ = R_5_ = H, R_4_ = B
Forsythiaside K	R_1_ = Rha, R_2_ = B, R_3_ = R_4_ = H, R_5_ = OCH_3_

Rha, Rhamnose; Api, apiose; Xyl, Xylose.

## Pharmacological activities

### Cardiovascular protection

Forsythiaside A and forsythiaside B can reduce cardiovascular disease damage and exert cardiovascular protection due to their extensive anti-inflammatory and antioxidant effects. Intravenous injection of 5–20 mg/kg forsythoside B dose-dependently reduced polymorph nuclear leukocyte (PMN) infiltration and myeloperoxidase (MPO) activity in a rat model of myocardial ischemia-reperfusion injury (7). The former releases inflammatory factors to damage cardiomyocytes after being activated by ischemic injury, while the latter is considered to be related to the occurrence of cardiovascular disease (8, 9). High-mobility group box 1 (HMGB1) is an inflammatory mediator released by necrotic cells or activated innate immune cells and can activate the NF-κB signaling pathway (10). Forsythoside B could attenuate the expression of HMGB1 and NF-κB in myocardial tissue, decrease the levels of troponin-T, TNF-α, and IL-6 in serum, and reduce the severity of myocardial injury (7). After 40 mg/kg of forsythiaside A was injected intraperitoneally into heart failure mice, the protein expression of NF-κB was decreased, and the levels of inflammatory factors TNF-α, IL-6, and IL-1β in serum were reduced, displaying the cardioprotective effect of forsythiaside A (11).

Nuclear factor-erythroid 2-related factor 2 (Nrf2) is a cytoprotective factor that can regulate the expression of genes encoding antioxidant, anti-inflammatory, and other related proteins. It could bind to antioxidant response elements (AREs) to initiate the transcription of downstream antioxidant genes (12). Forsythiaside A alleviated oxidative stress in mice with heart failure by activating the Nrf2/heme oxygenase-1 (HO-1) signaling pathway (11). Forsythiaside B reduced the content of MDA in the serum of myocardial ischemia-reperfusion rats, and reverse the decrease of SOD and GPx activities caused by myocardial ischemia (7).

Besides, forsythoside A showed the vasoprotective effect through relaxing the isolated rat aorta. Specifically, forsythiaside A dose-dependently inhibited norepinephrine-induced vasoconstriction by reducing the influx of extracellular calcium ions caused by norepinephrine (13). When low-density lipoprotein is oxidized and accumulated on the arterial wall for a long time, it will cause arteriosclerosis. Forsythoside B could reverse the cytotoxicity elicited by oxidized low-density lipoprotein, inhibit the *in vitro* oxidation of low-density lipoprotein to play a cytoprotective role (14).

### Anti-inflammation

Inflammation is a defense mechanism that occurs when tissues are stimulated by some damages such as trauma, infection by pathogens including bacteria, viruses, parasites, and other inflammatory agents. Inflammatory mediators released from activated mast cells, neutrophils, and macrophages serve as modulator to promote or inhibit the inflammation process (15–17). TLR4/NF-κB is a vital anti-inflammatory pathway, which has attracted much attention in numerous studies on the anti-inflammatory activity of forsythiasides.

Toll-like receptors are a family of pattern-recognition receptors mostly expressed on the surface of cells involved in innate immunity. As a Type I integral membrane protein, TLRs consist of two major parts: an ectodomain of leucine-rich repeats and a cytoplasmic domain of Toll/IL-1R homology domain (18). Among the ten different TLRs discovered in humans, TLR4 is well-known for its characteristic to detect lipopolysaccharide (LPS). LPS non-covalently associates with TLR4 to form an activated heterodimer (LPS/TLR4/myeloid differentiation-2) complex, assisted by membrane cluster differentiation-14 coreceptor (19). Then the complex dimerization recruits MyD88-adaptor-like protein and MyD88, which allows activation of several IL-1 receptor-associated kinases (IRAKs). These IRAKs lead to the ubiquitination of TNF-α receptor-associated factor 6 (TRAF-6), an adaptor molecule activating the transforming growth factor beta-activated kinase 1 (TAK1). Then TAK1 phosphorylates several kinases, resulting in NF-κB being released with other elements (20). However, unlike the above-mentioned TLR4/MyD88/NF-κB pathway, TLR4 can stimulate the production of type I interferons (IFN) as a result of acting on related proteins and phosphorylation of interferon regulatory factor 3 (IRF3) (20).

**FIGURE 2:**
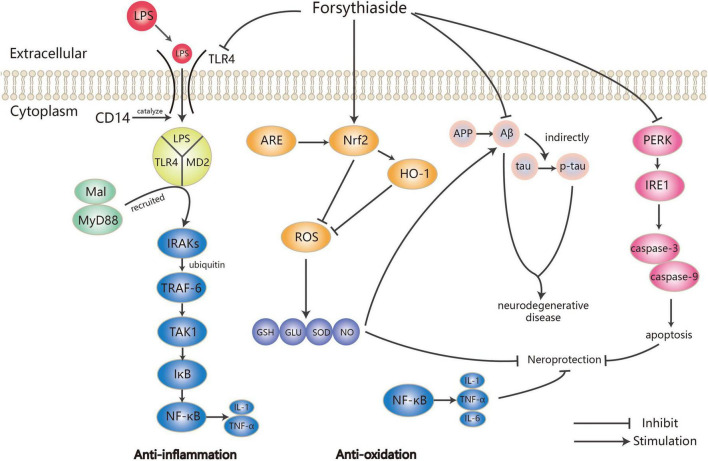
Mechanism of anti-inflammation and anti-oxidation by forsythiasides.

In dozens of studies on the anti-inflammatory effects of forsythosides A and B, LPS has been widely used to establish inflammation models. Forsythiaside A could significantly reverse the increase of TLR4 and NF-κB protein expression induced by LPS (21), which was also observed in the inflammation of PC12 cells induced by hypoxia/reoxygenation (22). Not only that, forsythosides A and B also reduced the expression of NF-κB through other pathways such as JNK-interacting protein (JIP)/c-Jun N-terminal kinase (JNK)/NF-κB and Nrf2/HO-1/NF-κB, thereby affecting the process of inflammation (23, 24). It is worth mentioning that although Nrf2 and NF-κB are the two key transcription factors regulating cellular response to oxidative stress and inflammation, respectively, the absence of Nrf2 can exacerbate NF-κB activity, leading to the increase of cytokine production (25). Both forsythiasides A and B could inhibit the expression of NF-κB by activating the Nrf2/HO-1 pathway, thereby preventing LPS-induced inflammation in BV2 microglia cells and RAW 264.7 macrophages as well as ovalbumin (OVA)-induced allergic airway inflammation in mice (24, 26–28). As a result, inflammatory factors such as TNF-α and ILs were also regulated in the inflammation model treated by forsythiasides. Forsythiasides could protect against inflammation *via* the NF-κB pathway by regulating TLR4 and Nrf2. Figure 2 shows how forsythiasides act on the TLR4/MyD88/NF-κB pathway to exert the anti-inflammatory effects. The anti-inflammation properties of forsythiasides A and B are presented in Table 2.

**TABLE 2:** Anti-inflammatory effects of forsythiasides.

Forsythiaside Subtype	Dose	Inflammation model	Action	Mechanism	References
A	20, 80, 320 μg/ml	LPS-induced RAW264.7 cells and primary lymphocytes	Increase of cell viability	Inhibition of HMGB1/TLR4/NF-κB pathway and downregulation of Foxp3, IL-10 and TGF-β1	21
A	1.25, 2.5, 5 μmol/L	Ischemia reperfusion-induced PC12 cells	Reduction of inflammatory response	Inhibition of TLR4/NF-κB pathway	22
B	10, 40 mg/kg	APP/PS1 mice	Decrease of Aβ deposition and tau phosphorylation, and reverse of cognitive decline	Attenuating the activation of JIP3/JNK and WDFY1/TLR3, inhibiting the NF-κB pathway	23
A	2.5, 5, 10 μg/ml	LPS-induced BV-2 cells	Reduction of inflammatory response	Regulating NF-κB and Nrf2/HO-1 pathways, reducing the release of TNF-α, IL-1β and PGE_2_	24
A	15, 30, 60 mg/kg	OVA-induced asthma in mice	Attenuating lung histopathology and suppressing inflammatory responses in asthma	Activation of Nrf2/HO-1 pathway, and decrease of IL-4, IL-5, and IL-13 levels	26
A and B	5, 10, 20 and 12.5, 25, 50, 100 μmol/L	LPS-induced RAW264.7 cells	Alleviation of inflammatory response	Activation of KEAP1/Nrf2/HO-1 pathway, reduction of NO, IL-1β, IL-6 and TNF-α levels	27, 28
A	80 μmol/L	Aβ_25–35_-treated hippocampal slices	Alleviation of learning and memory deficits	Suppressing the overexpression of COX-2 and MAGL proteins and upregulating the levels of 2-AG	33
A	60 mg/kg	LPS-induced acute lung injury in mice	Ameliorating pathological damage and macrophage infiltration of lung	Regulation of miR-124/CCL2 pathway	38
A	12.5, 25, 50 mg/kg	LPS-induced acute lung injury in mice	Attenuating inflammatory cell infiltration and pulmonary interstitial edema	Inhibiting TXNIP/NLRP3 pathway	90
A	20 mg/kg	Influenza A Virus-infected mice	Reducing lung inflammation and inflammatory cell infiltration	Regulation of RLRS-mediated pathways in lung immune cells	72
A	20, 40, 80 mg/kg	IBV-induced infectious bronchitis in avian	Ameliorating clinical signs and lung damage	Increasing CD3^+^, CD4^+^, CD8^+^ T lymphocytes, regulating IL-2,IL-4, IFN-α	78
A	15, 30 mg/kg	OVA-induced asthma in mice	Attenuating airway inflammatory cell infiltration	Depression of p38 MAPK/NF-κB pathway	91
A and B	30, 60, 120 μmol/L	CuSO_4_-induced Zebrafish	Relieving damage to the neuromasts in the lateral line	Reducing the expression of ROS, NO, Wdr3, and MRPs7	92
A	2.5, 5, 10 μg/ml	*S. aureus*-induced primary bovine mammary epithelial cells	Reduction of inflammatory response	Inhibition of MAPK/NF-κB pathways, down-regulation of the expression of TNF-α, IL-1β, IL-6	93
A	30 mg/kg	Chick type II collagen induced rheumatoid arthritis in mice	Relieving symptoms in rheumatoid arthritis	Decreasing the expression level of TNF-α protein in serum	94
A	30, 60 mg/kg	LPS-induced spleen of chicken	Reduction of inflammatory response	Suppressing the gene and protein levels of IL-17 and IL-6	95
A	40 mg/kg	Zymosan-induced peritonitis in mice	Alleviation of acute peritonitis	Deceasing the expression of NF-κB, the number of neutrophils and the release of TNF-α, IL-6 and MCP-1	96
A	25, 50, 100 μg/ml	LPS-induced human airway epithelial cells	Reduction of inflammatory response	Reduction of NO secretion and SOD level	97
A	5, 20, 80 mg/kg	2,4-dinitrochlorobenzene in ethanol induced ulcerative colitis in rats	Alleviation of colon lesions	Inhibiting the release of TNF-α and IL-2, increasing the expression of IL-4	98
A and B	6 mg/kg	Dimethylbenzene-induced ear swelling in mice	Alleviation of ear swelling and inflammatory response	Decreasing the production of TNF-α and IL-6	99
B	0.65, 1.30, 2.60 μmol/L	Caecal ligation and puncture -induced sepsis in rats	Reduction of lethality and counteraction of LPS activity	Reducing the levels of TNF-α, IL-6, HGMB1 and TREM-1	100

#### Neuroprotection

Alzheimer’s disease (AD) is an age-related neurodegenerative disease, whose pathological features are the neurofibrillary tangles formed by the deposition of amyloid-β (Aβ), hyperphosphorylated tau protein, and neuron loss (29). Among them, Aβ is a 38-43 amino acid polypeptide processed from amyloid precursor protein (APP), which plays an important role in causing the imbalance of synaptic homeostasis and clearance dysfunction of lysosomal (30, 31). Recently, deposition was reported to be promoted by interferon-induced transmembrane protein 3, a γ-secretase modulatory protein induced by inflammatory cytokines.

The neuroprotective effect of forsythiasides is related to their anti-neuroinflammatory activity. Specifically, they could significantly down-regulate inflammatory factors such as interleukin and tumor necrosis factor. The levels of inflammatory factors TNF-α, IL-1β, and IL-6 in the brain tissue of APP/PS1 mice and SAMP8 mice were higher than those of wild-type mice. Interestingly, forsythiasides could reverse these enhanced inflammatory factors. Furthermore, forsythiaside B inhibited NF-κB to exert anti-neuroinflammatory effect by insulting the activation of JIP-3/JNK and reducing the expression of WDFY1/TLR3 (32). Besides, forsythiaside A inhibited the cannabinoid receptor 1 (CB1R)-dependent NF-κB signaling pathway to reduce the secretion of TNF-α and PEG_2_ in organotypic hippocampal slices of mice (33).

In addition, forsythiasides increased the content of acetylcholine in Aβ-induced AD mouse, which is one of the important neurotransmitters in the central cholinergic system, indicating that forsythiasides have the potential to improve the function of acetylcholine (34).

#### Lung protection

Acute lung injury (ALI) is one of the earliest and highest morbidity complications after severe trauma or infection. Up-regulation of inflammatory factors serves as a sign of direct response and the development of sustained cell damage (35). A large number of studies have shown that forsythiasides A and B have protective effects on LPS-induced ALI. After the ALI mouse model was treated with forsythiasides, the pathological damage of the lung tissue was relieved in varying degrees (36, 37). At the same time, the gene and protein expression of TLR4, MyD88, and NF-κB were suppressed (36), and the levels of inflammatory factors such as TNF-α, IL-1β, and IL-6 were down-regulated (37). Furthermore, forsythiaside A could reduce the production of chemotactic protein CCL-2, inhibit the adhesion and migration of monocytes to type II lung epithelial cells, thereby decreasing the pathological symptoms of acute lung injury (38). In addition, forsythiaside A could reduce the inflammatory injury of H9N2 avian influenza virus-induced ALI mice by inhibiting MyD88 and NF-κB signaling pathways to down-regulate inflammatory factors such as TNF-α, IL-6, IL-1β (39, 40). Moreover, in mice with cigarette smoke-induced chronic obstructive pulmonary disease, forsythoside A not only decreased inflammatory cytokines and NO production by inhibiting NF-κB signaling pathway, but also increased glutathione/glutathione ratio of peptide disulfides that prevent lung damage (41).

#### Kidney protection

Nephrotic syndrome (NS) is a common type of glomerular disease in children and adults and has many complications resulting in difficult prognosis. Forsythiaside A significantly reduced the levels of urine protein, blood creatinine and urea nitrogen in a nephropathy rat model induced by adriamycin, and decreased the number of apoptotic cells in the kidney tissue (42). Forsythiaside A also dose-dependently depressed the expression of NF-κB p65/Macrophage inflammatory protein-2 in the renal tissue and the levels of inflammatory factors such as IL-6, IL-1β, and TNF-α in serum, improving survival in rats with doxorubicin-induced nephropathy (42).

Diabetic nephropathy (DN) is a common cause of mortality in diabetic patients, and is generally caused by persistent high glucose (HG). Podocytes play an important role in maintaining glomerular structure and filtration. Due to the limited division ability, the damage and reduction of podocytes are the key factors to accelerate the progression of DN. It was found that forsythiaside A inhibited matrix metalloproteinase-12, down-regulated extracellular regulated protein kinases (ERK)/p38 mitogen-activated protein kinase (MAPK) signaling pathway, reduced the expression levels of p-ERK, p-p38, and p-JNP in cells, and inactivated MAPK signaling to alleviate the oxidative stress and inflammation caused by HG (43).

#### Liver protection

Galactosamine (GalN) is a hepatotoxic substance that can inhibit the synthesis of RNA and protein in liver cells, causing diffuse liver necrosis and inflammation. In a mouse model of acute liver injury induced by LPS/GalN, forsythiaside A inhibited NF-κB activation and reduced TNF-α levels in serum, protecting against liver damage (44).

#### Bone protection

Osteoclasts, one of the components of bone tissue, have the function of bone resorption. They cooperate with osteoblasts to play a key role in the development and formation of bone (45). Forsythiaside A suppressed LPS-induced skull osteolysis in mice by decreasing the differentiation and formation of osteoclasts to restrain bone resorption. Besides, forsythiaside A also inhibited osteoclast differentiation and reduce ovariectomy-elicited bone loss in mice (46).

### Anti-oxidation

Nuclear factor-erythroid 2-related factor 2 plays a vital role in transcriptional activation of genes driven by the antioxidant responsive element (ARE), which mediates transcriptional regulation of phase II detoxification enzyme and antioxidant proteins to clear reactive oxygen species (ROS) (12). HO-1, one of Nrf2-target genes, exerts protection against oxidative injury and modulation of inflammation as well as contribution to angiogenesis (47). In the LPS-induced RAW 264.7 macrophage model, oxidative stress was observed (28). Forsythiaside A inhibited the activation of phosphatidylinositol 3-kinase (PI3K)/protein kinase B (AKT) pathway. At the same time, it activated the Nrf2/HO-1 pathway and reduced the level of ROS. Forsythoside A also depressed the production of inflammatory mediators NO and PGE_2_, and decreased the expression of pro-inflammatory cytokines TNF-α and IL-1β (28). Meanwhile, the regulatory effect of forsythiaside A on the Nrf2/HO-1 pathway was observed in the OVA-induced mouse asthma model (26). Figure 1 displays how forsythiasides exerts the anti-oxidation effects by regulation of the Nrf2/HO-1 pathway.

#### Free radicals scavenging

Free radicals are the product of the body’s oxidation process, and the appropriate number of free radicals helps maintain the body’s normal physiological functions. The body can maintain the free radical level in a stable range by scavenging the excessive free radicals. Too high concentration of free radicals in the body acts on lipids to undergo peroxidation. The resulting peroxide product MDA denatures cross-link proteins, damages DNA, inactivates enzymes and hormones to damage cells, and finally accelerates aging (48). Forsythiaside A exerted neuroprotective and hepatoprotective effects by clearing MDA (44, 49). Moreover, it was found that forsythiaside A had a dose-dependent anti-oxidant effect on scavenging DPPH free radicals, hydroxyl free radicals, and superoxide anion free radicals, etc. (50–52).

Superoxide dismutase (SOD) is a free radical scavenger widely present in aerobic metabolizing cells, which plays a key role in the body’s oxidation and antioxidant balance. Studies have shown that when inflammation occurs, the level of ROS in cells increases significantly (28). Forsythiaside A could strengthen the body’s antioxidant capacity, and relieve oxidative stress caused by inflammation or ischemic damage through enhancing the activity of cell SOD and increasing the content of glutathione (GSH), glutathione peroxidase (GSH-Px) and catalase (53, 54). The mechanism of action was related to regulation of the PI3K/AKT/Nrf2/HO-1 signaling pathway (28, 55), indicating that forsythiaside A has a certain potential in alleviating inflammation and oxidative damage.

#### Neuroprotection

There is certain evidence that excessive oxidative stress factors can produce toxicity to nerve cells, which is related to degenerative memory impairment in AD patients. SOD and GSH-Px are endogenous antioxidant enzymes, which reflect the body’s ability to scavenge free radicals and play a key role in the antioxidant capacity of brain cells. Forsythoside A exerted neuroprotective effects by activating Nrf2 and endoplasmic reticulum stress pathway to reduce cerebral ischemic damage. Forsythiaside A increased the activities of SOD and GSH-Px in the Aβ-induced aging mouse model, and reduced the levels of peroxidation product MDA and important oxygen free radicals NO *in vivo* and *in vitro*, alleviating learning and memory deficits in aging mice (49). Forsythiaside A also had a certain effect on delaying the body’s aging by increase of the antioxidant capacity.

#### Skin protection

Cell protective enzymes such as SOD and GPX can prevent excessive levels of active oxygen to maintain the body’s stability. Forsythiaside B had strong antioxidant and free radical scavenging activities. It activated the expression of Nrf2, regulated the level of nuclear transcription factors of cell protective enzymes, and induced the protective ability of phase II cells to exert skin protection (56). Transient receptor potential vanilloid 3 (TRPV3) channel plays an important role in skin physiology, which is associated with genetic Olmsted syndrome characterized by palmoplantar keratoderma and severe pruritus. Forsythiaside B had the therapeutic potential for the treatment of chronic pruritus, skin allergies or inflammation-related skin diseases due to its specific inhibition of overactive TRPV3. It suppressed channel currents activated by TRPV3 agonists in a dose-dependent manner and significantly attenuated acute and chronic pruritus in a dry skin mouse model (57). Another report showed that forsythiaside B had a protective effect on free radical-induced functional endothelial injury (58).

#### Liver protection

In the acute liver injury mouse model induced by LPS/GalN, forsythiaside A significantly reduced the content of MDA and attenuated the pathological injury of the mouse liver by activation of the Nrf2/HO-1 signaling pathway, indicating its liver protection effect related to anti-oxidation (44).

### Anti-bacterial activity

Forsythiasides A, B, and I show a widely anti-bacterial activity in many investigations. For instance, enrofloxacin, one of the most used fluoroquinolones, has good therapeutic effect on many pathogens such as *Escherichia coli*, Pseudomonas and *Aeromonas hydrophila*. Owing to the widespread use of quinolones, the bacterial resistance has become more and more prominent (59). Studies have found that forsythiaside A could down-regulate the expression of key genes in the differentiation family of drug-resistant nodular cells, and significantly inhibit enrofloxacin resistance to *A. hydrophila* (60, 61). *Forsythia suspensa* affected the growth of drug-resistant bacteria of *A. hydrophila*, presumably mainly through amino acid metabolism, glycolysis, carbon, and nitrogen metabolism, as well as stress-related ABC transport and chemotaxis pathways. Bacterial drug resistance is closely related to its efflux pump system, which can be reduced through inhibiting the activity or reducing the expression for efflux pump.

In addition, forsythiaside A has an obvious antibacterial activity against *E. coli*, *Staphylococcus aureus*, and *Streptococcus pneumoniae*. The K-B paper disk method to measure the diameter of the inhibition zone showed its successive decrease effect on the three bacteria (62). *In vivo* and *in vitro* experiments suggested that forsythiaside A had certain inhibitory effect on *S. aureus*, *Streptococcus lactis*, and *Streptococcus agalactiae* (63). Similarly, forsythiaside B was also found to have antibacterial activity against *Proteus mirabilis* and *S. aureus* (64). There are also many reports that forsythiaside A had strong inhibitory effect on *E. coli*, *Pseudomonas aeruginosa*, and *Bacillus subtilis* [32](65, 66). Moreover, forsythoside B displayed strong antibacterial activity against multi-drug resistant *S. aureus* (67). Although forsythiasides have extensive antibacterial effects, the related mechanism needs to be further studied. The antibacterial properties of forsythiasides are summarized in Table 3.

**TABLE 3:** Antibacterial effects of forsythiasides.

Forsythiaside subtype	Bacteria	References
A	Inhibiting the growth of *Escherichia coli*, *Staphylococcus aureus*, *Streptococcus pneumoniae*, *Bacillus subtilis*, *Streptococcus agalactiae*, *Pseudomonas aeruginosa*, and reversing the resistance of *Aeromonas hydrophila* to enrofloxacin	61–63, 65, 66, 101
B	Suppressing the growth of *Proteus mirabilis*, *Staphylococcus aureus*, multi-drug resistant *Staphylococcus aureus*	64, 67

### Anti-viral activity

As a commonly used Chinese medicine, *F. suspensa* has strong antiviral activity. Studies have exhibited that forsythiaside has inhibitory effect on a variety of viruses. Forsythiaside A could directly kill chicken infectious bronchitis viruses *in vitro* and inhibit the infection of infectious bronchitis viruses in a dose-dependent manner, but no obvious effect was observed in the infected cells. When a large dose of forsythiaside A was used to pretreat chicken embryo kidney cells, the infectivity of infectious bronchitis virus was significantly inhibited (68). The mechanism was probably related to the induction of IFN-α by forsythiaside A, which up-regulated the relevant factors of the janus kinase–signal transducer and activator of transcription signaling pathway (68). Further research found that forsythiaside A significantly inhibited the replication of the viruses in the cells, and enhance the expression of mRNA related to receptors such as melanoma differentiation-associated gene-5 laboratory of genetics and physiology 2, nod-like receptor family caspase activation and recruitment domain containing 5 and antiviral proteins such as IRF7, IFN-α, and IFN-β (69). The above evidence shows that forsythiaside A has the effect of anti-infectious bronchitis.

Matrix protein 1 (M1 protein), the most abundant one in viruses, plays an important role in maintaining the virus structure and the process of virus replication assembly, and germination. Forsythiaside A could cause slow or abnormal germination of influenza A viruses and the mechanism may relate to decrease in the expression of M1 protein and interfere with the germination process of newly formed viruses (70). Forsythiaside A alleviated the symptoms of weight loss and lung tissue damage caused by influenza virus infection in mice by attenuating the mRNA expression of TLR7, MyD88, IRAK4, TRAF6 in TLR7 signaling pathway and NF-κB p65, reducing the gene and protein expression of retinoicacidinduciblegene-1, mitochondrial antiviral signaling protein and NF-κB, and inhibiting the replication of influenza A viruses to control infection (71, 72). In addition, studies have found that forsythosides C and D were two main components against influenza viruses in the anti-influenza capsules of Forsythiae Fructus (73). It can be seen that forsythiasides have great potential in anti-virus.

### Immunomodulation

Regulatory T cells (Tregs), a subgroup of T cells, are important immune cells with independent functions in the body. Tregs not only participate in cellular immunity, but also maintain the body’s immune balance by suppressing effector T cells to avoid excessive immunity. Therefore, Tregs play a huge role in preventing autoimmune diseases, anti-graft rejection, and tumor immunity (74). The cell-specific nuclear transcription factor Foxp3 can characteristically mark Tregs and play a key role in the development and functional maintenance of Tregs (75).

Forsythiaside A was found to possess immunomodulatory effect by regulating Tregs (76). When the body is infected by endotoxin, endotoxemia will result. Bacterial endotoxin can increase the survival and proliferation ability of Tregs, and enhance their immunosuppressive function to inhibit the body’s immune response. Forsythiaside A exerted the immunomodulatory effect by reducing the level of peripheral blood Tregs and the expression of foxp3 transcription factors in endotoxin mice (21). In addition, forsythiaside A inhibited the replication of bovine viral diarrhea viruses in peripheral blood mononuclear cells cultured *in vitro*. It plays an important role in immune regulation by promoting the transcription of OX40, 4-1BB, and 4-1BBL which increase the regulation of the activation and proliferation of T cells (77). In addition, forsythiaside A significantly enhanced the number of CD3^+^, CD4^+^, CD8^+^ T lymphocytes in the blood of chickens infected with infectious bronchitis viruses (IBV), showing regulatory effect on the immune function of chickens (78).

### Antipyretic activity

The hypothalamus is the advanced center of body temperature regulation, and some nerve nuclei play an important role in this process, such as the paraventricular nucleus (PVN) and supraoptic nucleus. Furthermore, there are abundant temperature-sensing neurons in the periphery such as the dorsal root ganglia (DRG), which also exert a key role in thermoregulation. The temperature-sensitive protein TRPA1 is abundantly expressed in the body temperature regulation center (79). Forsythiaside A effectively reduced the body temperature of fever mice induced by subcutaneous injection of yeast suspension through increasing the expression of TRPA1 in the PVN, supraoptic nucleus and DRG (80). Not only that, forsythiaside A also inhibited the secretion of inflammatory mediators such as PGE2 and IL-8, and mitigated symptoms of fever in mice injected with yeast suspension subcutaneously. The mechanism of action may be related to inhibiting the expression of TRPV1 and reducing calcium influx and MAPK phosphorylation (81). Forsythiaside I, the main component of Qingqiao, was also found to possess the function of clearing heat and removing toxicity (82).

### Other activities

As active ingredients from a multifunctional medicinal plant, forsythiasides display an anticancer activity to a certain degree. Forsythia Fructus could prolong the survival time of melanoma mice, whose components forsythiasides A, E, and I significantly inhibited the cell viability of B16-F10 cells (83). In addition, forsythoside B suppressed the proliferative activity of cervical cancer cells by blocking the expression of NF-κB and up-regulating p21 binding to the cyclin E/CDK2 complex (84).

Besides, forsythiaside A significantly improved the survival rate of rats with cerebral ischemia and reduced neurological deficits by inhibiting neuronal apoptosis and attenuating the expression of caspase-3 and caspase-9 (85). Forsythiaside A obviously increased hair density and thickness in mice with androgenetic alopecia by 50 and 30%, respectively, and decreased the expression of caspase-9 and caspase-3 in the skin by 40 and 53%, respectively. It also inhibited DHT-induced apoptosis of human hair dermal papilla cells and human keratinocytes. Forsythiaside A is a natural product with the potential to treat androgenetic alopecia due to its protective effect on hair loss by inhibiting apoptosis and delaying hair cells entering the degenerative phase (86). Forsythiaside A attenuated APAP-induced hepatocyte degeneration and necrosis by inhibiting the PI3K/AKT pathway associated with apoptosis and reversing the abnormal expression of caspase-3, caspase-8, caspase-9, bax, and bcl-2 (87).

## Prospective and conclusion

As one of commonly used Chinese medicines, Forsythiae Fructus has extensive pharmacological effects, such as anti-inflammatory, clearing heat, removing toxicity, antibacterial, and antioxidant. Forsythiasides are abundant in *F. suspensa*. This article summarized the pharmacological activities of forsythiasides A-K (Table 4), which have a similar chemical structure. However, most of the current investigations focus on forsythiasides A and B. NF-κB is a DNA-binding transcription factor existing in eukaryotic cells, which participates in normal physiological processes such as immune and inflammatory response, and is seen as the convergence point of multiple signal pathways. The NF-κB immune signal pathway is closely related to the biological processes of development, proliferation, differentiation, and apoptosis in immune cells, and plays an important role in the regulation of inflammatory cytokine gene expression. In addition, studies have shown that the NF-κB signaling pathway is closely related to the occurrence of cancer (88). Forsythiasides exert anti-inflammatory, anti-tumor, neuroprotective and lung damage protective effects by inhibiting the activation of NF-κB signaling pathway. Furthermore, forsythiasides can also perform the pharmacological activity through activating the Nrf2 signaling pathway and down-regulated the expression of HMGB1 and TLR4. Many documents reported that the strong neuroprotective effect of forsythiasides is related to reduction of Aβ deposition, alleviation of inflammation and oxidative stress of nerve cells, and decrease of caspase-3 activation to inhibit cell apoptosis. Forsythiasides have potential to treat AD by reversing nerve damage and memory dysfunction.

**TABLE 4:** Pharmacological activities of forsythiasides.

Forsythiaside subtype	Pharmacological activity
A	Anti-inflammation, antivirus, antioxidation, immune regulation, antibacteria, abatement of fever, antitumor, neuroprotection, kidney tissue protection, lung tissue protection, liver tissue protection, hair loss protection, inhibition of osteoclast differentiation, inhibition of vasoconstriction
B	Anti-inflammation, antibacteria, cell protection, neuroprotection, lung tissue protection, skin protection, cardiomyocyte protection, anti-tumor
C	Antivirus
D	Antivirus
E	Antitumor
F	Still not clear
G	Still not clear
H	Still not clear
I	Abatement of fever, antibacteria
J	Still not clear
K	Still not clear

Although forsythiasides possess many pharmacological activities, there exist some limitations. Forsythiasides A and B show strong antibacterial activity and the ability to scavenge free radicals *in vitro*. In addition to normal bacteria, forsythiasides also have inhibitory effect on drug-resistant bacteria. However, the current investigations on antibacterial and free radical scavenging effects of forsythiasides are mostly concentrated on *in vitro* experiments, failing to further validate these *in vivo*. Forsythiasides interfere with the replication process of the virus, exhibiting the evident antiviral activity. However, the current literature only reported their inhibitory effects on chicken infectious bronchitis viruses and influenza A viruses. In addition, forsythiasides A and B were considered to be closely related to the pseudo-allergic reaction caused by Shuanghuanglian injection (89). However, there are few toxicology investigations reported.

In summary, subsequent studies on forsythiasides should be carried out in view of the above limitations. The NF-κB signaling pathway is closely relevant to a variety of human diseases such as inflammation, tumor, and tissue damage. The other pharmacological effects should be explored further based on NF-κB signaling pathway. Forsythiasides have potential to be an adjuvant drug for the treatment of Alzheimer’s disease owing to the significant neuroprotective effect, so their anti-AD mechanism should be explored further. The antibacterial and antioxidant activities of forsythiasides *in vivo* should also be investigated and relevant toxicological studies need to be carried out. Finally, it is hoped that the above-mentioned problems will be solved further and more evidence is provided for the clinical applications of forsythiasides.

## Author contributions

HZ, MJ, and LZ: conceptualization. H-XY and Q-PL: writing-original. H-XY and HZ: writing-review and editing. HZ, MJ, LZ, Y-XZ, Y-YC, PA, and Y-ZX: verification and recommendation. All authors contributed to the article and approved the submitted version.

## Conflict of interest

The authors declare that the research was conducted in the absence of any commercial or financial relationships that could be construed as a potential conflict of interest.

## Publisher’s note

All claims expressed in this article are solely those of the authors and do not necessarily represent those of their affiliated organizations, or those of the publisher, the editors and the reviewers. Any product that may be evaluated in this article, or claim that may be made by its manufacturer, is not guaranteed or endorsed by the publisher.
